# Guarding Reflex Inhibition Training Reduces Postoperative Urinary Retention After Urethral Bulking for Stress Urinary Incontinence: A Retrospective Single-Center Study

**DOI:** 10.3390/jcm14217701

**Published:** 2025-10-30

**Authors:** Nicole Fleischmann, Marlena Plagianos, Rachel Meiselman, Katherine Panushka

**Affiliations:** 1 White Plains Hospital Center, 41 East Post Road, White Plains, NY 10601, USA; 2 Montefiore/Albert Einstein College of Medicine, 1825 Eastchester Rd, Bronx, NY 10461, USA

**Keywords:** stress urinary incontinence, postoperative urinary retention, urethral bulking, lower urinary tract dysfunction, external urethral sphincter, guarding reflex

## Abstract

**Background/Objectives:** Postoperative urinary retention (POUR) occurs in 10–15% of women after surgical procedures for stress urinary incontinence (SUI). Guarding Reflex Inhibition Training (GRIT) is a novel behavioral approach that teaches patients to consciously inhibit involuntary pelvic floor contraction. We evaluated whether preoperative GRIT is associated with decreased POUR after urethral bulking with polyacrylamide hydrogel (PAHG). **Methods:** We performed a retrospective review of 145 women with SUI. Perioperative care was consistent across groups, separated by date of service; only those treated after November 2023 received structured GRIT instruction. The primary outcome was POUR, defined as the need for >1 episode of straight catheterization or discharge with a catheter. **Results:** POUR occurred in 15/106 (14.2%) patients without GRIT and 0/39 (0%) patients with GRIT. This difference was statistically significant (Fisher’s exact test *p* = 0.012), corresponding to an absolute risk reduction of 14.2% (95% CI: 4.8–23.9) and a number needed to treat (NNT) of 7. Post hoc power was >90%. **Conclusions:** Preoperative GRIT, a novel and reproducible training paradigm, was associated with a reduction in POUR following urethral bulking. By targeting conscious inhibition of the guarding reflex, GRIT highlights the potential for integrating behavioral retraining with procedural therapy across incontinence interventions.

## 1. Introduction

Urinary incontinence (UI) is one of the most common pelvic floor disorders, affecting over 60% of women and imposing major quality-of-life and economic burdens [1,2]. Stress urinary incontinence (SUI), often conceptualized as a structural failure, is managed with pelvic floor exercises to strengthen weak tissues or with surgical procedures to provide urethral support, such as midurethral slings. However, many women with SUI demonstrate coexisting neuromuscular impairments—including deficits in posture, balance, and proprioception—which coincide with intrinsic sphincter deficiency. These observations highlight the need for therapeutic approaches that not only correct anatomic defects but also enhance sphincter function and global neuromotor control [3].

Urodynamic investigations in women with SUI often reveal underlying voiding dysfunction, including incomplete relaxation or active urethral contraction during the micturition phase [4]. Classic studies show the most frequent diagnostic pattern is detrusor underactivity (DU) with reliance on abdominal strain to void [5,6]. Many women with SUIexhibit postural maneuvers to compensate for impaired temporal coordination of the pelvic floor muscles, suggesting a cortico-pelvic disconnection—a failure of sensorimotor integration whereby cortical intent to void cannot effectively down-regulate the guarding reflex at the level of the pelvic outlet [7,8,9,10].

Conservative management typically includes lifestyle modification and pelvic floor muscle exercises, particularly those aimed at strengthening the outlet [11]. However, population-based studies demonstrate that for urinary incontinence (UI) and lower urinary tract symptoms (LUTs), behavioral retraining to correct maladaptive toilet habits such as habitual holding, delayed voiding, and straining—may be a more effective than training to increase the force of contraction [12,13,14]. Pelvic floor muscle inhibition and relaxation are central to developing pelvic floor awareness, yet most patients never learn these skills outside of specialized physical therapy settings. Notably, women who undergo surgery without adequate pelvic floor awareness face a higher risk for recurrence and adverse outcomes, including postoperative urinary retention (POUR) [15].

Proprioception of the pelvic outlet muscles to initiate voiding is a developmental cognitive skill [16]. Lower urinary tract dysfunction (LUTD) is common in children, especially after toilet training has commenced [17]. Although numerous etiologies for functional obstruction have been proposed—including adverse childhood events, genetic variability, and alterations in the bladder microbiome—the shared result is often delayed or incomplete sensorimotor integration of bladder and outlet function [18,19]. Urodynamic studies in children with bladder and bowel dysfunction show staccato voiding and compensatory postural maneuvers to facilitate release [20]. Thus, dysfunctional elimination behaviors acquired in childhood may persist as latent maladaptive patterns resurfacing as urinary incontinence in adulthood [21,22,23].

Standard urotherapy (SU), as defined by the International Children’s Continence Society, comprises non-invasive, non-pharmacological interventions for lower urinary tract dysfunction (LUTD) and includes education, timed voiding schedules, bladder diaries, hydration, dietary guidance, and posture training. A key aim of SU is to ensure appropriate and coordinated pelvic muscle relaxation during voiding [24,25]. Despite the high prevalence of dysfunctional voiding in women with SUI, similar structured training programs have not yet been widely applied to adult women.

GRIT is an author-developed protocol that extends standard urotherapy to explicitly target guarding-reflex inhibition through sniff-enhanced respiration and posture sequencing (Figure A2). To our knowledge, this is the first study to apply a structured guarding reflex inhibition protocol in the perioperative management of women undergoing treatment for SUI. GRIT incorporates the core elements of SU—education, lifestyle changes, motor awareness, self-monitoring and reinforcement—while adding explicit training in awareness and inhibition of involuntary or voluntary pelvic floor contraction and tone.

To test the potential impact of GRIT on voiding dysfunction, we evaluated postoperative urinary retention (POUR) rates in women receiving a urethral bulking procedure for SUI. We hypothesized that GRIT would reduce the incidence of POUR by addressing the outlet dysfunction that interferes with normal voiding initiation.

## 2. Materials and Methods

Study Design and Setting: We conducted a single-center retrospective cohort study of women who elected distal urethral injection of polyacrylamide hydrogel (PAHG) at White Plains Hospital Center between 1 November 2022, and 15 March 2024. Before November 2023, patients received pre-procedure conservative management with behavioral therapy, which focused on healthy toileting habits. Beginning in November 2023, GRIT was codified and incorporated into a urotherapy protocol which could be adopted as standard care. Group assignment was nonrandomized and based on date of procedure.

Institutional review board approval was obtained from Montefiore Medical Center (IRB# 2021-13157), which granted a waiver of informed consent due to the retrospective nature of the study.

Participants: Eligible patients were women with SUI or mixed urinary incontinence (MUI) and:

1. Over the age of 18.
2. Pelvic organ prolapse ≤ II in any compartment.
3. Demonstration of SUI on physical examination or urodynamic evaluation.
4. Pre-procedure post-void residual (PVR) less than 150 mL.
5. Negative pregnancy test at time of procedure.
6. No prior surgical or injectable procedure for SUI within 3 months of treatment date.
7. Self-reported stress or stress-predominant leakage that is bothersome enough to warrant treatment regardless of frequency or severity of leakage episodes.

Exclusion criteria included:

1. Concomitant surgery for pelvic organ prolapse at time of intervention.
2. Active urinary tract infection or asymptomatic bacteriuria at pre-procedure screening.
3. Neurogenic lower urinary tract dysfunction (LUTD) due to spinal cord injury, multiple sclerosis etc.
4. Uncontrolled bleeding risk or active anti-coagulation, with the exception of prophylactic-dose aspirin (81 mg daily).

**Behavioral therapy and GRIT Protocol:**

**Figure A1:**
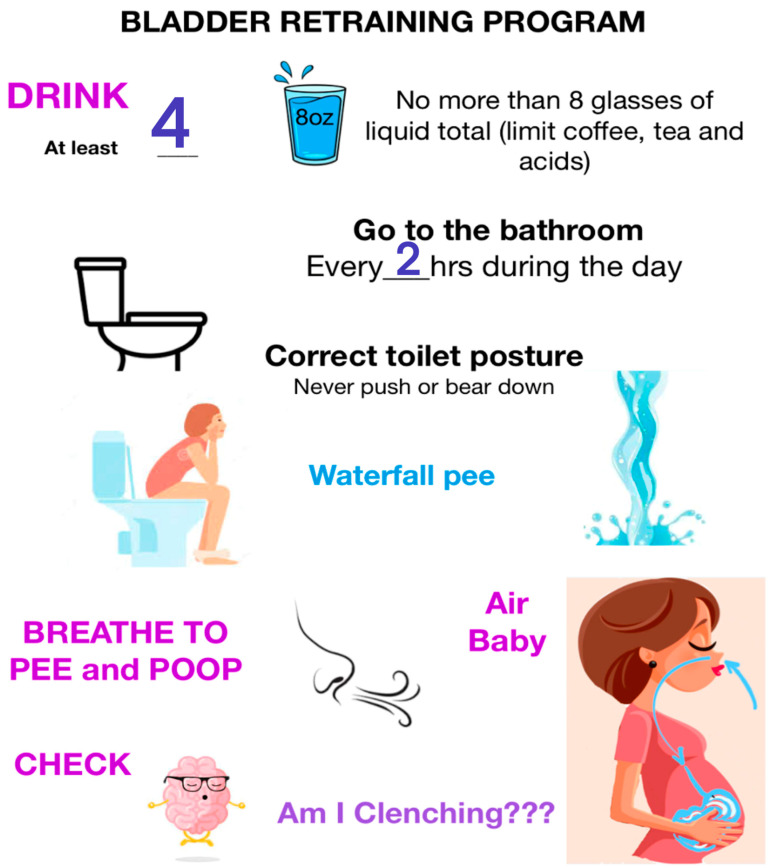
Patient handout for standard behavioral therapy.

**Figure A2:**
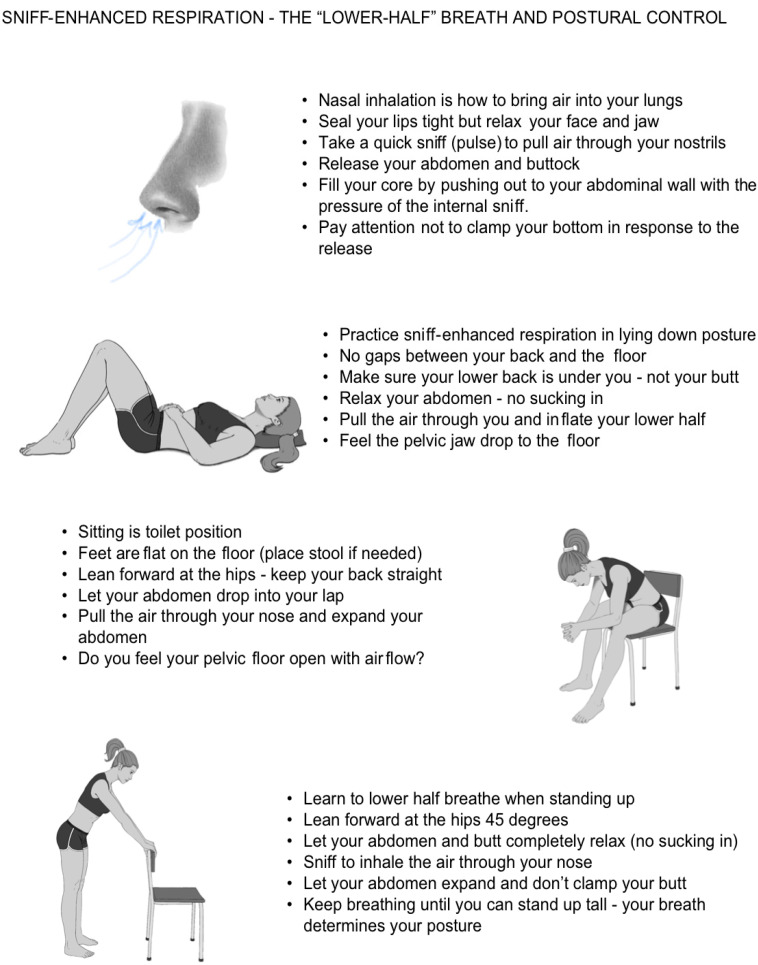
Handout provided during Guarding Reflex Inhibition Training (GRIT): GRIT is introduced to patients during a brief, ~15 min instructional session. The same sniff-enhanced respiration (SER) cue is practiced across three functional postures—supine, seated/toileting, and upright—using consistent core principles: a neutral, elongated spine and active release of abdominal and pelvic muscle tension. The protocol reframes voiding as an active neuromotor skill by training cortical awareness and volitional inhibition of pelvic overactivity.

All patients received written educational material on healthy voiding mechanics—including bowel regulation, timed voiding, and avoidance of straining—consistent with conventional behavioral therapy [18,23]. See Figure A1.

**Group 1: No GRIT—Standard behavioral therapy (November 2022–March 2023):**

(1) Fluid Management: Drinking fluid (32 to 64 oz) evenly throughout the day.
(2) Timed voiding: Voiding every 2 to 3 h (except during sleep) to discourage holding behaviors as well as “just in case” voiding.
(3) Posture training: Assuming a seated and leaning-forward posture for urination and defecation.
(4) Relaxation: Facilitation of voiding and defecation by breathing with the diaphragm instead of bearing down or pushing.
(5) Mindful body awareness: Practicing recognition of involuntary pelvic floor tightening in response to outside stimuli (i.e., stressful situation) or inner stimulus (i.e., heightened emotion).
(6) Limitation: No specific training on reflex inhibition was provided.

**GROUP 2: Behavioral therapy with GRIT (November 2023–March 2024):**

Patients received above education plus structured GRIT instruction. GRIT integrates sniff-enhanced respiration with posture sequencing to retrain outlet relaxation. The protocol was delivered consistently as follows:

1. Initial instruction: A 15 min in-office training session at the preoperative visit, including demonstration of the GRIT sequence and provision of reinforcement handouts.
2. Breathing initiation: Training with a volitional sniff to activate diaphragmatic descent (sniff-enhanced respiration, SER) and trigger awareness of pelvic release.
3. Posture sequencing: SER was practiced across three functional positions—supine, seated/toileting, and upright—each emphasizing a neutral spine and abdominal release.
4. GRIT: learning to identify and inhibit daily involuntary guarding reactions using deliberate SER.
5. Integration: Repetition of the sniff-posture sequence across positions reinforced cortical awareness, voluntary inhibition of the guarding reflex, and functional voiding mechanics.
6. Pre-procedure review: Check for understanding and a brief refresher immediately prior to injection procedure.

### 2.1. Surgical Procedure

All procedures were performed by a single surgeon in an ambulatory setting with minimal sedation. The technique for distal urethral injection of polyacrylamide hydrogel (PAHG) under cystoscopic guidance is described by Clearwater et al. [26]. Approximately 3 mL PAHG was injected until visible coaptation was achieved without overcorrection. Hemostasis and urethral patency were confirmed endoscopically, and a 12 French catheter was placed to drain the bladder at the conclusion of the procedure.

POUR was defined as the need for more than one episode of straight catheterization in the recovery room. Voiding trials were passive starting from an empty bladder and the decision to perform catheterization was based on patient comfort (feeling of fullness) or bladder scan reading of 400 mL or greater. If the patient went home with an indwelling catheter or returned to clinic later for catheterization, this was also considered POUR.

### 2.2. Statistical Analysis

Continuous variables were summarized as means ± standard deviations; categorical variables as frequencies and percentages. Group comparisons used chi-square or Fisher’s exact tests. Logistic regression assessed the association between GRIT participation and POUR, adjusting for concomitant pelvic reconstruction. *p* < 0.05 was considered statistically significant.

## 3. Results

### 3.1. Baseline Characteristics

Baseline characteristics were broadly similar between groups (Table A1). GRIT patients were younger on average (mean age 54.3 vs. 60.0 years, *p* = 0.036) and less often postmenopausal (39% vs. 63%, *p* = 0.008). Other factors—including body mass index (BMI), smoking status, diabetes, prior SUI treatment, and concomitant overactive bladder (OAB) therapy—were comparable across groups. Baseline characteristics are summarized in Table A1.

### 3.2. Primary Outcome—POUR

Among 145 women who underwent urethral bulking, 39 received perioperative GRIT instruction and 106 did not. No patients in the GRIT group developed postoperative urinary retention (POUR), compared with 15 cases (14.2%) in the non-GRIT group (*p* = 0.012). In multivariable logistic regression, GRIT showed a protective effect with an adjusted odds ratio (AOR) of 12.0 (95% CI: 0.65–250, *p* = 0.094), though this did not reach statistical significance after controlling for covariates due to wide confidence intervals.

Key covariates associated with POUR included:

- Concomitant surgery: strongly associated with POUR (46.7% vs. 9.4%, *p* = 0.0008; AOR 5.1, 95% CI: 1.45–17.9, *p* = 0.011).
- Postmenopausal status: more common among those with POUR (86.7% vs. 51.6%, *p* = 0.0097), though not significant in adjusted models (AOR 3.53, 95% CI: 0.75–16.7, *p* = 0.11).
- Diabetes mellitus: higher prevalence in POUR (20% vs. 4.7%, *p* = 0.021; AOR 9.1, 95% CI: 1.33–61.9, *p* = 0.025).

Age trended higher among patients with POUR (mean 64.9 vs. 57.3 years, *p* = 0.055). BMI, smoking status, recurrent UTI, prior SUI treatment, mixed UI, and OAB therapy were not significantly associated with POUR.

Women who developed POUR were older compared with those who did not (mean age 64.9 vs. 57.6 years, *p* = 0.072). The proportion of postmenopausal women was also higher among those with POUR (86.7% vs. 52.3%, *p* = 0.011). However, when adjusted for other covariates, neither age nor postmenopausal status remained independently associated with POUR (age: NS; postmenopausal status: AOR 2.7, 95% CI 0.65–11.5, *p* = 0.17).

Body mass index was similar between groups (27.1 vs. 28.4 kg/m^2^, *p* = 0.48). Co-surgery was strongly associated with POUR, occurring in nearly half of women with retention (46.7% vs. 10.8%, *p* = 0.0015), and remained significant in multivariate analysis (AOR 5.8, 95% CI 1.73–19.5, *p* = 0.0044). Diabetes (20.0% vs. 4.6%, *p* = 0.052), recurrent urinary tract infections (33.3% vs. 15.4%, *p* = 0.081), and prior SUI treatment (46.7% vs. 25.4%, *p* = 0.063) were more frequent in women with POUR, though these associations did not achieve statistical significance after adjustment. No significant group differences were observed with respect to smoking status, history of pelvic radiation, mixed urinary incontinence, or prior overactive bladder treatment.

## 4. Discussion

### 4.1. Postoperative Urinary Retention (POUR)

POUR is a common complication of incontinence surgery, with reported rates averaging 20% after midurethal slings (MUS) [26,27,28,29]. Retention is typically transient (24–48 h) and managed with self-catheterization or indwelling Foley catheter. Compared with MUS, POUR after urethral bulking is both lower in frequency (5–10%) and less likely to require surgical intervention. Notably, 1 to 3% of MUS procedures resulting in POUR will go on to require sling lysis or loosening [27,28,29,30]. While the majority of cases resolve within 24 h, even transient retention contributes to morbidity, health care utilization, and patient dissatisfaction [31,32].

In this cohort, POUR occurred in 14.2% of women who did not undergo GRIT, consistent with published retention rates for urethral bulking procedures [26,27,28]. By contrast, no cases occurred in the GRIT group, highlighting the potential of structured reflex-inhibition training to mitigate perioperative retention. Although multivariate models were underpowered, the protective signal for GRIT was consistent, and co-surgery emerged as the strongest independent risk factor for POUR, in line with prior literature identifying concomitant procedures as contributors to transient retention [31].

We included (in Table A2) comparisons of the associations between POUR and treatment intervention (GRIT), as well as with each of the items presented in Table A1. First we examined the relationships in bivariate analyses, then entered each item that was statistically significant (*p* < 0.05) into a multivariate regression. The model included treatment group (GRIT or no GRIT), co-surgery, menopausal status, and diabetes. Although the multivariate regression found no statistical association at the 0.05 level between treatment group and POUR, the *p*-value was 0.09, and the estimated odds ratio of 12 suggests a large protective effect of GRIT. This indicates the study was too underpowered to demonstrate statistical significance when adjusting for covariates, but a larger trial may confirm the observed effect. Importantly, the study did have adequate power to demonstrate a difference in treatment and POUR in unadjusted analyses.

### 4.2. POUR as Physiologic Marker Rather than Passive Obstruction

Although the mechanism by which bulking agents treat SUI remains speculative, prevailing theories suggest they narrow the urethral lumen, thereby increasing resistance to urine flow [32,33]. This same resistance, while protective against urine leakage, may also contribute to temporary resistance to voiding directly after injection therapy.

In contrast, the mechanism for POUR after midurethral slings likely differs from bulking agents as they are intentionally placed tension free to avoid direct compression of the urethra. Some investigators propose that valsalva voiding can induce a functional obstruction after MUS even in women with no preoperative emptying difficulties [27,28].

In this study, we focus on distal injection, which is delivered closer to the external sphincter complex (EUS), an area known to have potential neuromuscular dysfunction in women with SUI [4,34]. The observed reduction in postoperative urinary retention (POUR) among women trained with GRIT suggests that retention at the level of the EUS may represent a deficit in pelvic floor relaxation more than a purely mechanical obstruction [34]. Similarl to POUR after MUS, retention after distal injection may arise from underlying LUTD such as detrusor underactivity and valsalva voiding, both of which are well established contributors to POUR [27,33,34,35].

From this perspective, POUR can be viewed as a physiologic marker of outlet dysfunction. The inability or difficulty relaxing and releasing the pelvic floor after SUI procedures is a likely a neuromuscular phenomenon rather than solely a consequence of passive obstruction. Pushing behaviors may enable flow under normal conditions but become less effective when urethral outlet resistance increases after injection. GRIT provides an alternative pathway, presumably by decreasing outlet tone with volitional inhibition of the guarding reflex, facilitating coordinated muscular control of the urethral outlet and pelvic floor.

#### Comparative Context in Behavioral Therapy

Prior behavioral interventions for SUI and bladder outlet dysfunction have focused largely on muscle activation or strengthening. The OPAL randomized controlled trial showed that intensive pelvic floor muscle training improved continence, but the protocol emphasized contraction rather than coordinated relaxation [36]. Similarly, Lazaros et al. reported that pelvic floor muscle training improved symptoms of functional bladder outlet obstruction, but again through a strengthening paradigm [37]. Even when adjunctive modalities such as electrical stimulation and biofeedback are used, the primary emphasis is upon enhanced activation and not on training patients to inhibit maladaptive reflexes [38].

In addition, a recent Cochrane systematic review of behavioral interventions for urinary incontinence in women concluded that pelvic floor muscle training and related strategies improve continence outcomes but highlighted that available protocols overwhelmingly emphasize strengthening and do not address mechanisms of relaxation or reflex inhibition [39]. Importantly, the review noted that existing trials assessed symptom improvement, post-void residuals, and quality-of-life measures, but none evaluated perioperative endpoints such as postoperative urinary retention. To our knowledge, this is the first study to evaluate a structured behavioral protocol specifically for POUR prevention after an SUI procedure.

### 4.3. Study Limitations

We conducted this study during the period prior to our practical implementation of Guarding Reflex Inhibition Training (GRIT), representing a window of time when patients were treated without this intervention. We developed the protocol to address the observation that women cognitively understood the goal to relax to void, but were unable to effectively move their voluntary muscles into a relaxed/lengthened position, a demonstration of diminished proprioceptive skills. Since that time, GRIT has become an integral part of our treatment algorithm, precluding the possibility of a future comparison group without it. While this limits study design, it also provides a unique opportunity to evaluate outcomes before and after the implementation of GRIT.

The retrospective, time-based group design introduces potential for selection bias. Non-GRIT procedures were performed earlier in the study period. Although the surgeon was beyond the learning curve at study onset, temporal differences may still represent a variable—such as subtle changes in injection technique or perioperative practice patterns—that could influence outcomes. Importantly, the complete absence of POUR in the GRIT group, despite this potential variability, suggests that the observed effect is unlikely to be explained by operator factors alone.

We adjusted for demographic and clinical variables, but unmeasured confounders may have contributed to the observed differences. Baseline differences in age and menopausal status between groups could also have affected retention risk, although neither remained significant in adjusted analyses. Co-surgery emerged as the strongest independent risk factor for POUR, consistent with prior literature, but our sample size limited the ability to explore potential interactions with GRIT.

Finally, we did not employ imaging, functional brain mapping, EMG, or urodynamic measures to directly confirm reductions in pelvic floor activity or improvements in detrusor–sphincter coordination. Validated questionnaires assessing severity of leakage or quality of life were also not used, as the primary outcome was POUR rather than treatment effect or subjective cure. Follow-up was therefore limited to the perioperative period, and our findings apply specifically to immediate voiding outcomes rather than long-term continence.

### 4.4. Clinical Directions

The significant results of this single-center retrospective study warrant validation in a prospective randomized controlled trial. Future work should also incorporate mechanistic studies using functional neuroimaging or electrophysiologic techniques to directly confirm changes in cortical and subcortical pathways involved in guarding reflex inhibition and volitional pelvic floor control. Surface or needle EMG may provide a means to quantify reductions in pelvic tone during voiding, while pelvic ultrasound could noninvasively assess levator ani lengthening during SER, a process that often fails to occur with verbal cues to “just relax” [40].

GRIT reframes continence as the ability to inhibit rather than merely strengthen sphincter activity, aligning well with other neurological rehabilitation models that emphasize conscious suppression of maladaptive reflexes [41,42]. By embedding outlet awareness and volitional release into urotherapy, GRIT provides a reproducible perioperative protocol that reduces POUR and complements targeted sphincter therapies such as bulking, slings, or emerging regenerative approaches [43,44].

As sphincter-targeted interventions evolve—including regenerative bulking agents and stem cell-based treatments—the patient’s ability to inhibit involuntary pelvic floor reflexes will become increasingly important to optimize the outcomes of stress urinary incontinence (SUI) surgeries. It is our clinical recommendation that GRIT be further studied so that alongside the routine prescription of pelvic floor strengthening exercises (Kegels), patients are also trained to pay attention to toileting behaviors and posture to ensure a balance of contraction and effective relaxation during elimination of their bladder and bowels. These lessons can be feasibly integrated into routine preoperative counseling as a brief, office-based exercise to reduce POUR risk and potentially, distal bulking agent efficacy.

## 5. Conclusions

Guarding reflex inhibition training (GRIT) is a codified neuromuscular training program that uses physiologic breathing mechanics (sniff-enhanced respiration) to facilitate pelvic floor relaxation, enabling patients to overcome voluntary or involuntary guarding during the initiation of urine flow. This study explored whether GRIT could reduce postoperative urine retention after distal urethral injection and whether reflex-inhibition training could improve perioperative outcomes. The significant reduction in POUR among GRIT-trained women suggests that retention reflects a combination of maladaptive voiding patterns and transient mechanical obstruction. POUR may therefore serve as a physiologic marker of modifiable outlet dysfunction.

Taken together, these findings suggest that transient POUR after urethral bulking is not simply a complication, but an opportunity to identify underlying neuromuscular dysfunction. By combining behavioral retraining with structural intervention, patients educated with GRIT had less postoperative retention, highlighting the role of cortical–subcortical control in continence. Prospective, multicenter randomized trials incorporating physiologic endpoints and longer-term follow-up are warranted to validate efficacy and durability. If confirmed, GRIT could represent both a practical perioperative tool and a scalable conservative therapy, bridging the gap between urotherapy, targeted sphincter therapy, and surgical intervention in female SUI.

## Data Availability

The data presented in this study are available on reasonable request from the corresponding author. The data are not publicly available due to privacy restrictions.

## Appendix A

**Table A1:** Baseline Characteristics.

	GRIT (*N* = 39)	No GRIT (*N* = 106)	Overall (*N* = 145)	*p*-Value
Age				0.0087
Mean (SD)	52.9 (12.8)	60.0 (14.7)	58.1 (14.5)	
Median [Min, Max]	51.4 [21.0, 83.2]	56.8 [37.7, 96.8]	54.3 [21.0, 96.8]	
BMI				0.25
Mean (SD)	27.3 (6.28)	28.7 (6.89)	28.3 (6.74)	
Median [Min, Max]	26.0 [19.0, 43.0]	27.2 [15.8, 59.9]	27.0 [15.8, 59.9]	
Missing	0 (0%)	1 (0.9%)	1 (0.7%)	
Co-surgery	3 (7.7%)	16 (15.1%)	19 (13.1%)	0.28
Post Menopausal	14 (35.9%)	67 (63.2%)	81 (55.9%)	0.0033
Smoker				0.55
Never	31 (79.5%)	89 (84.0%)	120 (82.8%)	
Former	8 (20.5%)	15 (14.2%)	23 (15.9%)	
Current	0 (0%)	2 (1.9%)	2 (1.4%)	
Diabetes	4 (10.3%)	6 (5.7%)	10 (6.9%)	0.46
Recurrent UTI	4 (10.3%)	21 (19.8%)	25 (17.2%)	0.22
Pelvic Radiation	0 (0%)	0 (0%)	0 (0%)	>0.99
Prior SUI Treatment	9 (23.1%)	31 (29.2%)	40 (27.6%)	0.44
Mixed Urinary Incontinence	17 (43.6%)	64 (60.4%)	81 (55.9%)	0.071
Overactive Bladder Treatment	4 (10.3%)	17 (16.0%)	21 (14.5%)	0.38

Abbreviations: BMI = body mass index; UTI = urinary tract infection; SUI = stress urinary incontinence; GRIT = Guarding Reflex Inhibition Training.

**Table A2:** Illustrates POUR incidence across groups and subgroups.

	No POUR (*N* = 130)	POUR (*N* = 15)	Overall (*N* = 145)	Bivariate, (Unadjusted) *p*-Value	Multivariate, Adjusted OR, (95% CI), *p*-Value
Treatment Group				0.012	AOR 12.0 (0.65–250) *p* = 0.094
GRIT	39 (30.5%)	0 (0%)	39 (27.3%)		
No GRIT	89 (69.5%)	15 (100%)	104 (72.7%)		
Age				0.055	
Mean (SD)	57.3 (14.5)	64.9 (14.1)	58.1 (14.6)		
Median [Min, Max]	53.6 [21.0, 96.8]	64.2 [45.1, 91.4]	54.2 [21.0, 96.8]		
BMI				0.47	
Mean (SD)	28.4 (6.79)	27.1 (6.58)	28.3 (6.76)		
Median [Min, Max]	27.0 [15.8, 59.9]	25.0 [19.4, 42.0]	27.0 [15.8, 59.9]		
Missing	1 (0.8%)	0 (0%)	1 (0.7%)		
Co-surgery	12 (9.4%)	7 (46.7%)	19 (13.3%)	0.0008	AOR: 5.1 (1.45–17.9), *p* = 0.011
Post Menopausal	66 (51.6%)	13 (86.7%)	79 (55.2%)	0. 0097	AOR: 3.53 (0.75–16.7), *p* = 0.11
Smoker				>0.99	
Never	106 (82.8%)	13 (86.7%)	119 (83.2%)		
Former	20 (15.6%)	2 (13.3%)	22 (15.4%)		
Current	2 (1.6%)	0 (0%)	2 (1.4%)		
Diabetes	6 (4.7%)	3 (20.0%)	9 (6.3%)	0.021	AOR: 9.1 (1.33–61.9), *p* = 0.025
Recurrent UTI	20 (15.6%)	5 (33.3%)	25 (17.5%)	0.14	
Pelvic Radiation	0 (0%)	0 (0%)	0 (0%)	>0.99	
Prior SUI Treatment	33 (25.8%)	7 (46.7%)	40 (28.0%)	0.067	
MUI	70 (54.7%)	10 (66.7%)	80 (55.9%)	0.38	
OAB treatment	19 (14.8%)	2 (13.3%)	21 (14.7%)	>0.99	

Abbreviations: BMI = body mass index; UTI = urinary tract infection; SUI = stress urinary incontinence; GRIT = Guarding Reflex Inhibition Training.
